# Combining Semilattices and Semimodules

**DOI:** 10.1007/978-3-030-71995-1_6

**Published:** 2021-03-23

**Authors:** Filippo Bonchi, Alessio Santamaria

**Affiliations:** 8 University of Oxford, Oxford, UK; 9 Sorbonne Université - LIP6, Paris, France; Dipartimento di Informatica, Università degli Studi di Pisa, Pisa, Italy

**Keywords:** algebraic theories, monads, weak distributive laws

## Abstract

We describe the canonical weak distributive law $\delta :\mathcal S\mathcal P\rightarrow \mathcal P\mathcal S$ of the powerset monad $\mathcal P$ over the *S*-left-semimodule monad $\mathcal S$, for a class of semirings *S*. We show that the composition of $\mathcal P$ with $\mathcal S$ by means of such $\delta$ yields *almost* the monad of convex subsets previously introduced by Jacobs: the only difference consists in the absence in Jacobs’s monad of the empty convex set. We provide a handy characterisation of the canonical weak lifting of $\mathcal P$ to $\mathbb {EM}(\mathcal S)$ as well as an algebraic theory for the resulting composed monad. Finally, we restrict the composed monad to finitely generated convex subsets and we show that it is presented by an algebraic theory combining semimodules and semilattices with bottom, which are the algebras for the finite powerset monad $\mathcal P_f$.

## 1 Introduction

Monads play a fundamental role in different areas of computer science since they embody notions of computations [32], like nondeterminism, side effects and exceptions. Consider for instance automata theory: deterministic automata can be conveniently regarded as certain kind of coalgebras on $\mathbb{S}\mathrm{et}$ [33], nondeterministic automata as the same kind of coalgebras but on $\mathbb{EM}(\mathcal{P}_f)$ [35], and weighted automata on $\mathbb{EM}(\mathcal{S})$ [4]. Here, $\mathcal{P}_f$ is the finite powerset monad, modelling nondeterministic computations, while $\mathcal{S}$ is the monad of semimodules over a semiring $S$, modelling various sorts of quantitative aspects when varying the underlying semiring $S$. It is worth mentioning two facts: first, rather than taking coalgebras over $\mathbb{EM}(T)$, the category of algebras for the monad $T$, one can also consider coalgebras over $\mathbb{K}\mathrm{l}(T)$, the Kleisli category induced by $T$ [20]; second, these two approaches based on monads have lead not only to a deeper understanding of the subject, but also to effective proof techniques [6,7,14], algorithms [1,8,22,36,39] and logics [19,21,27].

Since compositionality is often the key to master complex structures, computer scientists devoted quite some efforts to *compose monads* [40] or the equivalent notion of *algebraic theories* [24]. Indeed, the standard approach of composing monads by means of *distributive laws* [3] turned out to be somehow unsatisfactory. On the one hand, distributive laws do not exist in many relevant cases:

* Supported by the Ministero dell'Università e della Ricerca of Italy under Grant No. 201784YSZ5, PRIN2017 – ASPRA (*Analysis of Program Analyses*).

see [28,41] for some no-go theorems; on the other hand, proving their existence is error-prone: see [28] for a list of results that were mistakenly assuming the existence of a distributive law of the powerset monad over itself.

Nevertheless, some sort of weakening of the notion of distributive law–e.g., distributive laws of functors over monads [26]–proved to be ubiquitous in computer science: they are GSOS specifications [38], they are sound coinductive up-to techniques [7] and complete abstract domains [5]. In this paper we will exploit *weak distributive laws* in the sense of [15] that have been recently shown successful in composing the monads for nondeterminism and probability [17].

The goal of this paper is to somehow combine the monads $\mathcal{P}_f$ and $\mathcal{S}$ mentioned above. Our interest in $\mathcal{S}$ relies on the wide expressiveness provided by the possibility of varying $S$: for instance by taking $S$ to be the Boolean semiring, one obtains the monad $\mathcal{P}_f$; by fixing $S$ to be the field of reals, coalgebras over $\mathbb{EM}(\mathcal{S})$ turn out be linear dynamical systems [34].

We proceed as follows. Rather than composing $\mathcal{P}_f$, we found it convenient to compose the *full*, not necessarily finite, powerset monad $\mathcal{P}$ with $\mathcal{S}$. In this way we can reuse several results in [12] that provide necessary and sufficient conditions on the semiring $S$ for the existence of a canonical weak [15] distributive law $\delta\colon \mathcal{SP} \to \mathcal{PS}$. Our first contribution (Theorem 21) consists in showing that such $\delta$ has a convenient alternative characterisation, whenever the underlying semiring is a *positive semifield*, a condition that is met, e.g., by the semirings of Booleans and non-negative reals.

Such characterisation allows us to give a handy definition of the *canonical weak lifting* of $\mathcal{P}$ over $\mathbb{EM}(\mathcal{S})$ (Theorem 24) and to observe that such lifting is *almost* the same as the monad $\mathcal{C}\colon \mathbb{EM}(\mathcal{S}) \to \mathbb{EM}(\mathcal{S})$ defined by Jacobs in [25] (Remark 25): the only difference is the absence in $\mathcal{C}$ of the empty subset. Such difference becomes crucial when considering the composed monads, named $\mathcal{CM}\colon \mathbb{S}\mathrm{et} \to \mathbb{S}\mathrm{et}$ in [25] and $\mathcal{P}_c\mathcal{S}\colon \mathbb{S}\mathrm{et} \to \mathbb{S}\mathrm{et}$ in this paper: the latter maps a set $X$ into the set of convex subsets of $\mathcal{S}X$, while the former additionally requires the subsets to be non-empty. It turns out that while $\mathbb{K}\mathrm{l}(\mathcal{CM})$ is not $\mathbb{CPPO}$-enriched, a necessary condition for the coalgebraic framework in [20], $\mathbb{K}\mathrm{l}(\mathcal{P}_c\mathcal{S})$ indeed is (Theorem 30).

Composing monads by means of weak distributive laws is rewarding in many respects: here we exploit the fact that algebras for the composed monad $\mathcal{P}_c\mathcal{S}$ coincide with $\delta$-algebras, namely algebras for both $\mathcal{P}$ and $\mathcal{S}$ satisfying a certain pentagonal law. One can extract from this law some distributivity axioms that, together with the axioms for semimodules (algebras for the monad $\mathcal{S}$) and those for complete semilattices (algebras for the monad $\mathcal{P}$), provide an algebraic theory presenting the monad $\mathcal{P}_c\mathcal{S}$ (Theorem 32).

We conclude by coming back to the finite powerset monad $\mathcal{P}_f$. By replacing, in the above theory, complete semilattices with semilattices with bottom (algebras for the monad $\mathcal{P}_f$) one obtains a theory presenting the monad $\mathcal{P}_{fc}\mathcal{S}$ of *finitely generated* convex subsets (Theorem 35), which is formally defined as a restriction of the canonical $\mathcal{P}_c\mathcal{S}$. The theory, displayed in Table 1, consists of the

**Table 1.** The sets of axioms $E_{\mathcal{SL}}$ for semilattices (left), $E_{\mathcal{LSM}}$ for $S$-semimodules (right) and $E_{\mathcal{D}'}$ for their distributivity (bottom).

| | | |
|---|---|---|
| $(x \sqcup y) \sqcup z = x \sqcup (y \sqcup z)$ | $(x + y) + z = x + (y + z)$ | $(\lambda +_S \mu) \cdot x = \lambda \cdot x + \mu \cdot x$ |
| $x \sqcup y = y \sqcup x$ | $x + y = y + x$ | $0_S \cdot x = 0$ |
| $x \sqcup \bot = x$ | $x + 0 = x$ | $(\lambda\mu) \cdot x = \lambda \cdot (\mu \cdot x)$ |
| $x \sqcup x = x$ | | $\lambda \cdot (x + y) = \lambda \cdot x + \lambda \cdot y$ |
| | | $\lambda \cdot 0 = 0$ |
| $\lambda \cdot \bot = \bot$ for $\lambda \neq 0_S$ | $\lambda \cdot (x \sqcup y) = (\lambda \cdot x) \sqcup (\lambda \cdot y)$ | |
| $x + \bot = \bot$ | $x + (y \sqcup z) = (x + y) \sqcup (x + z)$ | |

theory presenting the monad $\mathcal{P}_f$ and the theory presenting the monad $\mathcal{S}$ with four distributivity axioms.

To save space we had to omit most of the proofs of the results in this article: the interested reader can find them in [9].

**Notation.** We assume the reader to be familiar with monads and their maps. Given a monad $(M, \eta^M, \mu^M)$ on $\mathbb{C}$, $\mathbb{EM}(M)$ and $\mathbb{Kl}(M)$ denote, respectively, the Eilenberg-Moore category and the Kleisli category of $M$. The latter is defined as the category whose objects are the same as $\mathbb{C}$ and a morphism $f\colon X \to Y$ in $\mathbb{Kl}(M)$ is a morphism $f\colon X \to M(Y)$ in $\mathbb{C}$. We write $U^M\colon \mathbb{EM}(M) \to \mathbb{C}$ and $U_M\colon \mathbb{Kl}(M) \to \mathbb{C}$ for the canonical forgetful functors, and $F^M\colon \mathbb{C} \to \mathbb{EM}(M)$, $F_M\colon \mathbb{C} \to \mathbb{Kl}(M)$ for their respective left adjoints. Recall, in particular, that $F^M(X) = (X, \mu^M_X)$ and, for $f\colon X \to Y$, $F^M(f) = M(f)$. Given $n$ a natural number, we denote by $\underline{n}$ the set $\{1, \ldots, n\}$.

## 2 (Weak) Distributive laws

Given two monads $S$ and $T$ on a category $\mathbb{C}$, is there a way to compose them to form a new monad $ST$ on $\mathbb{C}$? This question was answered by Beck [3] and his theory of *distributive laws*, which are natural transformations $\delta\colon TS \to ST$ satisfying four axioms and that provide a canonical way to endow the composite functor $ST$ with a monad structure. We begin by recalling the classic definition. In the following, let $(T, \eta^T, \mu^T)$ and $(S, \eta^S, \mu^S)$ be two monads on a category $\mathbb{C}$.

**Definition 1.** *A* distributive law *of the monad $S$ over the monad $T$ is a natural transformation $\delta\colon TS \to ST$ such that the following diagrams commute.*

$$\begin{array}{ccccc} TSS & \xrightarrow{\delta S} & STS & \xrightarrow{S\delta} & SST \\ {\scriptstyle T\mu^S}\big\downarrow & & & & \big\downarrow{\scriptstyle \mu^S T} \\ TS & & \xrightarrow{\quad\delta\quad} & & ST \end{array} \qquad \begin{array}{ccccc} TTS & \xrightarrow{T\delta} & TST & \xrightarrow{\delta T} & STT \\ {\scriptstyle \mu^T S}\big\downarrow & & & & \big\downarrow{\scriptstyle S\mu^T} \\ TS & & \xrightarrow{\quad\delta\quad} & & ST \end{array} \tag{1}$$

$$\begin{array}{ccc} & T & \\ {\scriptstyle T\eta^S}\swarrow & & \searrow{\scriptstyle \eta^S T} \\ TS & \xrightarrow{\quad\delta\quad} & ST \end{array} \qquad \begin{array}{ccc} & S & \\ {\scriptstyle \eta^T S}\swarrow & & \searrow{\scriptstyle S\eta^T} \\ TS & \xrightarrow{\quad\delta\quad} & ST \end{array}$$

One important result of Beck's theory is the bijective correspondence between distributive laws, liftings to Eilenberg-Moore algebras and extensions to Kleisli categories, in the following sense.

**Definition 2.** *A* lifting *of the monad $S$ to $\mathbb{EM}(T)$ is a monad $(\tilde{S}, \eta^{\tilde{S}}, \mu^{\tilde{S}})$ where*

$$\begin{array}{ccc} \mathbb{EM}(T) & \xrightarrow{\tilde{S}} & \mathbb{EM}(T) \\ {\scriptstyle F^T}\uparrow & & \uparrow{\scriptstyle F^T} \\ \mathbb{C} & \xrightarrow{S} & \mathbb{C} \end{array} \quad \textit{commutes,} \quad U^T\eta^{\tilde{S}} = \eta^S U^T, \quad U^T\mu^{\tilde{S}} = \mu^S U^T.$$

*An* extension *of the monad $T$ to $\mathbb{K}\mathrm{l}(S)$ is a monad $(\tilde{T}, \eta^{\tilde{T}}, \mu^{\tilde{T}})$ such that*

$$\begin{array}{ccc} \mathbb{C} & \xrightarrow{T} & \mathbb{C} \\ {\scriptstyle F_S}\downarrow & & \downarrow{\scriptstyle F_S} \\ \mathbb{K}\mathrm{l}(S) & \xrightarrow{\tilde{T}} & \mathbb{K}\mathrm{l}(S) \end{array} \quad \textit{commutes,} \quad \eta^{\tilde{T}} F_S = F_S\eta^T, \quad \mu^{\tilde{T}} F_S = F_S\mu^T.$$

Böhm [11] and Street [37] have studied various weaker notions of distributive law; here we shall use the one that consists in dropping the axiom involving $\eta^T$ in Definition 1, following the approach of Garner [15].

**Definition 3.** *A* weak distributive law of $S$ over $T$ *is a natural transformation $\delta\colon TS \to ST$ such that the diagrams in* (1) *regarding $\mu^S$, $\mu^T$ and $\eta^S$ commute.*

There are suitable weaker notions of liftings and extensions which also bijectively correspond to weak distributive laws as proved in [11,15].

**Definition 4.** *A* weak lifting *of $S$ to $\mathbb{EM}(T)$ consists of a monad $(\tilde{S}, \eta^{\tilde{S}}, \mu^{\tilde{S}})$ on $\mathbb{EM}(T)$ and two natural transformations*

$$U^T\tilde{S} \xrightarrow{\iota} SU^T \xrightarrow{\pi} U^T\tilde{S}$$

*such that $\pi\iota = id_{U^T\tilde{S}}$ and such that the following diagrams commute:*

$$\begin{array}{ccccc} U^T\tilde{S}\tilde{S} & \xrightarrow{\iota\tilde{S}} & SU^T\tilde{S} & \xrightarrow{S\iota} & SSU^T \\ {\scriptstyle U^T\mu^{\tilde{S}}}\downarrow & & & & \downarrow{\scriptstyle \mu^S U^T} \\ U^T\tilde{S} & & \xrightarrow{\iota} & & SU^T \end{array} \qquad \begin{array}{ccc} & U^T & \\ {\scriptstyle U^T\eta^{\tilde{S}}}\swarrow & & \searrow{\scriptstyle \eta^S U^T} \\ U^T\tilde{S} & \xrightarrow{\iota} & SU^T \end{array} \tag{2}$$

$$\begin{array}{ccccc} SSU^T & \xrightarrow{S\pi} & SU^T\tilde{S} & \xrightarrow{\pi\tilde{S}} & U^T\tilde{S}\tilde{S} \\ {\scriptstyle \mu^S U^T}\downarrow & & & & \downarrow{\scriptstyle U^T\mu^{\tilde{S}}} \\ SU^T & & \xrightarrow{\pi} & & U^T\tilde{S} \end{array} \qquad \begin{array}{ccc} & U^T & \\ {\scriptstyle \eta^S U^T}\swarrow & & \searrow{\scriptstyle U^T\eta^{\tilde{S}}} \\ SU^T & \xrightarrow{\pi} & U^T\tilde{S} \end{array} \tag{3}$$

*A* weak extension *of $T$ to $\mathbb{K}\mathrm{l}(S)$ is a functor $\tilde{T}\colon \mathbb{K}\mathrm{l}(S) \to \mathbb{K}\mathrm{l}(S)$ together with a natural transformation $\mu^{\tilde{T}}\colon \tilde{T}\tilde{T} \to \tilde{T}$ such that $F_S T = \tilde{T}F_S$ and $\mu^{\tilde{T}}F_S = F_S\mu^T$.*

**Theorem 5 ([3,11,15]).** *There is a bijective correspondence between (weak) distributive laws $TS \to ST$, (weak) liftings of $S$ to $\mathbb{EM}(T)$ and (weak) extensions of $T$ to $\mathbb{K}\mathrm{l}(S)$.*

## 3 The Powerset and Semimodule Monads

**The Monad $\mathcal{P}$.** Let us now consider, as $S$, the *powerset* monad $(\mathcal{P}, \eta^{\mathcal{P}}, \mu^{\mathcal{P}})$, where $\eta^{\mathcal{P}}_X(x) = \{x\}$ and $\mu^{\mathcal{P}}_X(\mathcal{U}) = \bigcup_{U \in \mathcal{U}} U$. Its algebras are precisely the complete semilattices and we have that $\mathbb{K}\mathrm{l}(\mathcal{P})$ is isomorphic to the category $\mathbb{R}\mathrm{el}$ of sets and relations. Hence, giving a distributive law $T\mathcal{P} \to \mathcal{P}T$ is the same as giving an extension of $T$ to $\mathbb{R}\mathrm{el}$: for this to happen the notion of weak cartesian functor and natural transformation is crucial.

**Definition 6.** *A functor* $T \colon \mathbb{S}\mathrm{et} \to \mathbb{S}\mathrm{et}$ *is said to be* weakly cartesian *if and only if it preserves weak pullbacks. A natural transformation* $\varphi \colon F \to G$ *is said to be* weakly cartesian *if and only if its naturality squares are weak pullbacks.*

Kurz and Velebil [29] proved, using an original argument of Barr [2], that an endofunctor $T$ on $\mathbb{S}\mathrm{et}$ has at most one extension to $\mathbb{R}\mathrm{el}$ and this happens precisely when it is weakly cartesian; similarly a natural transformation $\varphi \colon F \to G$, with $F$ and $G$ weakly cartesian, has at most one extension $\tilde{\varphi} \colon \tilde{F} \to \tilde{G}$, precisely when it is weakly cartesian. The following result is therefore immediate.

**Proposition 7 ([15, Corollary 16]).** *For any monad* $(T, \eta^T, \mu^T)$ *on* $\mathbb{S}\mathrm{et}$*:*

1. *There exists a unique distributive law of* $\mathcal{P}$ *over* $T$ *if and only if* $T$*,* $\eta^T$ *and* $\mu^T$ *are weakly Cartesian.*
2. *There exists a unique* weak *distributive law of* $\mathcal{P}$ *over* $T$ *if and only if* $T$ *and* $\mu^T$ *are weakly Cartesian.*

**The Monad $\mathcal{S}$.** Recall that a *semiring* is a tuple $(S, +, \cdot, 0, 1)$ such that $(S, +, 0)$ is a commutative monoid, $(S, \cdot, 1)$ is a monoid, $\cdot$ distributes over $+$ and $0$ is an annihilating element for $\cdot$. In other words, a semiring is a ring where not every element has an additive inverse. Natural numbers $\mathbb{N}$ with the usual operations of addition and multiplication form a semiring. Similarly, integers, rationals and reals form semirings. Also the Booleans $\mathbb{B}\mathrm{ool} = \{0, 1\}$ with $\vee$ and $\wedge$ acting as $+$ and $\cdot$, respectively, form a semiring.

Every semiring $S$ generates a *semimodule* monad $\mathcal{S}$ on $\mathbb{S}\mathrm{et}$ as follows. Given a set $X$, $\mathcal{S}(X) = \{\varphi \colon X \to S \mid \mathit{supp}\, \varphi \text{ finite}\}$, where $\mathit{supp}\, \varphi = \{x \in X \mid \varphi(x) \neq 0\}$. For $f \colon X \to Y$, define for all $\varphi \in \mathcal{S}(X)$

$$\mathcal{S}(f)(\varphi) = \Big(y \mapsto \sum_{x \in f^{-1}\{y\}} \varphi(x)\Big) \colon Y \to S.$$

This makes $\mathcal{S}$ a functor. The unit $\eta^{\mathcal{S}}_X \colon X \to \mathcal{S}(X)$ is given by $\eta^{\mathcal{S}}_X(x) = \Delta_x$, where $\Delta_x$ is the Dirac function centred in $x$, while the multiplication $\mu^{\mathcal{S}}_X \colon \mathcal{S}^2(X) \to \mathcal{S}(X)$ is defined for all $\Psi \in \mathcal{S}^2(X)$ as

$$\mu^{\mathcal{S}}_X(\Psi) = \Big(x \mapsto \sum_{\varphi \in \mathit{supp}\, \Psi} \Psi(\varphi) \cdot \varphi(x)\Big) \colon X \to S.$$

**Table 2.** Definition of some properties of a semiring $S$. Here $a, b, c, d \in S$.

| | |
|---|---|
| Positive | $a + b = 0 \implies a = 0 = b$ |
| Semifield | $a \neq 0 \implies \exists x.\, a \cdot x = x \cdot a = 1$ |
| Refinable | $a + b = c + d \implies \exists x, y, z, t.\, x + y = a,\ z + t = b,\ x + z = c,\ y + t = d$ |
| (A) | $a + b = 1 \implies a = 0$ or $b = 0$ |
| (B) | $a \cdot b = 0 \implies a = 0$ or $b = 0$ |
| (C) | $a + c = b + c \implies a = b$ |
| (D) | $\forall a, b.\ \exists x.\, a + x = b$ or $b + x = a$ |
| (E) | $a + b = c \cdot d \implies \exists t \colon \{(x, y) \in S^2 \mid x + y = d\} \to S$ such that $\sum_{x+y=d} t(x, y)x = a, \quad \sum_{x+y=d} t(x, y)y = b, \quad \sum_{x+y=d} t(x, y) = c.$ |

An algebra for $\mathcal{S}$ is precisely a *left-$S$-semimodule*, namely a set $X$ equipped with a binary operation $+$, an element $0$ and a unary operation $\lambda\cdot$ for each $\lambda \in S$, satisfying the equations in Table 1. Indeed, if $X$ carries a semimodule structure then one can define a map $a \colon \mathcal{S}X \to X$ as, for $\varphi \in \mathcal{S}X$,

$$a(\varphi) = \sum_{x \in X} \varphi(x) \cdot x \tag{4}$$

where the above sum is finite because so is *supp* $\varphi$. Vice versa, if $(X, a)$ is an $\mathcal{S}$-algebra, then the corresponding left-semimodule structure on $X$ is obtained by defining for all $\lambda \in S$ and $x, y \in X$

$$x +^a y = a(x \mapsto 1, y \mapsto 1), \qquad 0^a = a(\varepsilon), \qquad \lambda \cdot^a x = a(x \mapsto \lambda). \tag{5}$$

Above and in the remainder of the paper, we write the list $(x_1 \mapsto s_1, \dots, x_n \mapsto s_n)$ for the only function $\varphi \colon X \to S$ with support $\{x_1, \dots, x_n\}$ mapping $x_i$ to $s_i$ and we write the empty list $\varepsilon$ for the function constant to 0. For instance, for $a = \mu^{\mathcal{S}}_X \colon \mathcal{S}\mathcal{S}X \to \mathcal{S}X$, the left-semimodule structure is defined for all $\varphi_1, \varphi_2 \in \mathcal{S}X$ and $x \in X$ as

$$(\varphi_1 +^{\mu^{\mathcal{S}}} \varphi_2)(x) = \varphi_1(x) + \varphi_2(x), \qquad 0^{\mu^{\mathcal{S}}}(x) = 0, \qquad (\lambda \cdot^{\mu^{\mathcal{S}}} \varphi_1)(x) = \lambda \cdot \varphi_1(x).$$

Proposition 7 tells us exactly when a (weak) distributive law of the form $T\mathcal{P} \to \mathcal{P}T$ exists for an arbitrary monad $T$ on $\mathbb{S}\mathrm{et}$. Take then $T = \mathcal{S}$: when are the functor $\mathcal{S}$ and the natural transformations $\eta^{\mathcal{S}}$ and $\mu^{\mathcal{S}}$ weakly cartesian? The answer has been given in [12] (see also [18]), where a complete characterisation in purely algebraic properties for $S$ is provided. In Table 2 we recall such properties.

**Theorem 8 ([12]).** *Let $S$ be a semiring.*

1. *The functor $\mathcal{S}$ is weakly cartesian if and only if $S$ is positive and refinable.*
2. *$\eta^{\mathcal{S}}$ is weakly cartesian if and only if $S$ enjoys* (A) *in Table 2.*
3. *If $\mathcal{S}$ is weakly cartesian, then $\mu^{\mathcal{S}}$ is weakly cartesian if and only if $S$ enjoys* (B) *and* (E) *in Table 2.*

*Remark 9.* In [12, Proposition 9.1] it is proved that if $S$ enjoys (C) and (D), then $S$ is refinable; if $S$ is a positive semifield, then it enjoys (B) and (E). In the next Proposition we prove that if $S$ is a positive semifield then it is also refinable, hence $\mathcal{S}$ and $\mu^{\mathcal{S}}$ are weakly cartesian.

**Proposition 10.** *If $S$ is a positive semifield, then it is refinable.*

*Proof.* Let $a$, $b$, $c$ and $d$ in $S$ be such that $a+b=c+d$. If $a+b=0$, then take $x=y=z=t=0$, otherwise take

$$x=\frac{ac}{c+d},\quad y=\frac{ad}{c+d},\quad z=\frac{bc}{c+d},\quad t=\frac{bd}{c+d}.$$

Then $x+y=a$, $z+t=b$, $x+z=c$, $y+t=d$. □

*Example 11.* It is known that, for $\mathcal{S}=\mathbb{N}$, a distributive law $\delta\colon \mathcal{SP}\to\mathcal{PS}$ exists. Indeed one can check that all conditions of Theorem 8 are satisfied, therefore we can apply Proposition 7.1. In this case, the monad $\mathcal{S}X$ is naturally isomorphic to the commutative monoid monad, which given a set $X$ returns the collection of all *multisets* of elements of $X$. The law $\delta$ is well known (see e.g. [15,23]): given a multiset $\langle A_1,\ldots,A_n\rangle$ of subsets of $X$ in $\mathcal{SP}X$, where the $A_i$'s need not be distinct, it returns the set of multisets $\{\langle a_1,\ldots,a_n\rangle \mid a_i\in A_i\}$.

**Convex Subsets of Left-semimodules.** Theorem 8 together with Proposition 7.1 tell us that whenever the element 1 of $S$ can be decomposed as a non-trivial sum there is no distributive law $\delta\colon \mathcal{SP}\to\mathcal{PS}$. Semirings with this property abound, for example $\mathbb{Q}$, $\mathbb{R}$, $\mathbb{R}^+$ with the usual operations of sum an multiplication, as well as $\mathbb{B}$ool (since $1\vee 1=1$). Such semirings are precisely those for which the notion of *convex subset* of their left-semimodules is non-trivial. For the existence of a *weak* distributive law, however, this condition on $1_S$ is not required: convexity will indeed play a crucial role in the definition of the weak distributive law.

**Definition 12.** *Let $S$ be a semiring, $X$ an $S$-left-semimodule and $A\subseteq X$. The* convex closure *of $A$ is the set*

$$\overline{A}=\left\{\sum_{i=1}^{n}\lambda_i\cdot a_i \mid n\in\mathbb{N},\ a_i\in A,\ \sum_{i=1}^{n}\lambda_i=1\right\}\subseteq X.$$

*The set $A$ is said to be* convex *if and only if $A=\overline{A}$.*

Recalling that the category of $S$-left-semimodules is isomorphic to $\mathbb{EM}(\mathcal{S})$, we can use (4) to translate Definition 12 of convex subset of a semimodule into the following notion of convex subset of a $\mathcal{S}$-algebra $a\colon \mathcal{S}X\to X$.

**Definition 13.** *Let $S$ be a semiring, $(X,a)\in\mathbb{EM}(\mathcal{S})$, $A\subseteq X$. The* convex closure *of $A$ in $(X,a)$ is the set*

$$\overline{A}^{a}=\left\{a(\varphi)\mid \varphi\in\mathcal{S}X,\, supp\,\varphi\subseteq A,\, \sum_{x\in X}\varphi(x)=1\right\}.$$

*$A$ is said to be* convex *in $(X, a)$ if and only if $A = \overline{A}^a$. We denote by $\mathcal{P}_c^a X$ the set of convex subsets of $X$ with respect to $a$.*

*Remark 14.* Observe that $\emptyset$ is convex, because $\overline{\emptyset}^a = \emptyset$, since there is no $\varphi \in \mathcal{S}X$ with empty support such that $\sum_{x\in X} \varphi(x) = 1$.

*Example 15.* Suppose $S$ is such that $\eta^{\mathcal{S}}$ is weakly cartesian (equivalently (A) holds: $x + y = 1 \implies x = 0$ or $y = 0$), for example $S = \mathbb{N}$, and let $(X, a) \in \mathbb{EM}(\mathcal{S})$. A $\varphi \in \mathcal{S}X$ such that $\sum_{x\in X} \varphi(x) = 1$ and $supp\,\varphi \subseteq A$ is a function that assigns 1 to *exactly one* element of $A$ and 0 to all the other elements of $X$. These functions are precisely all the $\Delta_x$ for those elements $x \in A$. Since $a\colon \mathcal{S}X \to X$ is a structure map for an $\mathcal{S}$-algebra, it maps the function $\Delta_x$ into $x$. Therefore $\overline{A}^a = \{a(\Delta_x) \mid x \in A\} = \{x \mid x \in A\} = A$. Thus *all* $A \in \mathcal{PS}X$ are convex.

*Example 16.* When $S = \mathbb{B}\text{ool}$, we have that $\mathcal{S}$ is naturally isomorphic to $\mathcal{P}_f$, the finite powerset monad, whose algebras are idempotent commutative monoids or equivalently semilattices with a bottom element. So, for $(X, a) \in \mathbb{EM}(\mathcal{S})$, a $\varphi \in \mathcal{S}X$ such that $\sum_{x\in X} \varphi(x) = 1$ and $supp\,\varphi \subseteq A$ is any finitely supported function from $X$ to $\mathbb{B}\text{ool}$ that assigns 1 to at least one element of $A$. Intuitively, such a $\varphi$ selects a non-empty finite subset of $A$, then $a(\varphi)$ takes the join of all the selected elements. Thus, $\overline{A}^a$ adds to $A$ all the possible joins of non-empty finite subsets of $A$: $A$ is convex if and only if it is closed under binary joins.

## 4 The Weak Distributive Law $\delta\colon \mathcal{SP} \to \mathcal{PS}$

Weak extensions of $\mathcal{S}$ to $\mathbb{K}\text{l}(\mathcal{P}) = \mathbb{R}\text{el}$ only consist of extensions of the functor $\mathcal{S}$ and of the multiplication $\mu^{\mathcal{S}}$, for which necessary and sufficient conditions are listed in Theorem 8. Hence for semirings $S$ satisfying those criteria a weak distributive law $\delta\colon \mathcal{SP} \to \mathcal{PS}$ does exist, and it is unique because there is only one extension of the functor $\mathcal{S}$ to $\mathbb{R}\text{el}$.

**Theorem 17.** *Let $S$ be a positive, refinable semiring satisfying* (B) *and* (E) *in Table 2. Then there exists a unique weak distributive law $\delta\colon \mathcal{SP} \to \mathcal{PS}$ defined for all sets $X$ and $\Phi \in \mathcal{SP}X$ as:*

$$\delta_X(\Phi) = \left\{ \varphi \in \mathcal{S}X \mid \exists \psi \in \mathcal{S}(\ni_X).\, \begin{cases} \forall A \in \mathcal{P}X.\, \Phi(A) = \sum\limits_{x\in A} \psi(A, x) & (a) \\ \forall x \in X.\, \varphi(x) = \sum\limits_{A\ni x} \psi(A, x) & (b) \end{cases} \right\} \tag{6}$$

*where $\ni_X$ is the set $\{(A, x) \in \mathcal{P}X \times X \mid x \in A\}$.*

The above $\delta$, which is obtained by following the standard recipe of Proposition 7, is illustrated by the following example.

*Example 18.* Take $S = \mathbb{R}^+$ with the usual operations of sum and multiplication. Consider $X = \{x, y, z, a, b\}$, $A_1 = \{x, y\}$, $A_2 = \{y, z\}$ and $A_3 = \{a, b\}$. Let $\Phi \in \mathcal{S}(\mathcal{P}X)$ be defined as

$$\Phi = (A_1 \mapsto 5, \quad A_2 \mapsto 9, \quad A_3 \mapsto 13)$$

and $\Phi(A) = 0$ for all other sets $A \subseteq X$, so $supp\,\Phi = \{A_1, A_2, A_3\}$. In order to find an element $\varphi \in \delta_X(\Phi)$, we can first take a $\psi \in \mathcal{S}(\ni_X)$ satisfying condition (a) in (6) and then compute the $\varphi \in \mathcal{S}X$ using condition (b).

Among the $\psi \in \mathcal{S}(\ni_X)$, consider for instance the following:

$$\psi = \begin{pmatrix} (A_1, x) \mapsto 2 & (A_2, y) \mapsto 4 & (A_3, a) \mapsto 6 \\ (A_1, y) \mapsto 3 & (A_2, z) \mapsto 5 & (A_3, b) \mapsto 7 \end{pmatrix}.$$

Since $\Phi(A_1) = \psi(A_1, x) + \psi(A_1, y)$, $\Phi(A_2) = \psi(A_2, y) + \psi(A_2, z)$ and $\Phi(A_3) = \psi(A_3, a) + \psi(A_3, b)$, we have that $\psi$ satisfies condition (a) in (6). Condition (b) forces $\varphi$ to be the following:

$$\varphi = (x \mapsto 2, \quad y \mapsto 3 + 4, \quad z \mapsto 5, \quad a \mapsto 6, \quad b \mapsto 7).$$

*Remark 19.* If $S$ enjoys (A) in Table 2, then the transformation $\delta$ given in (6) is actually a distributive law, and for $S = \mathbb{N}$ we recover the well-known $\delta$ of Example 11. Example 18 can be repeated with $S = \mathbb{N}$: then $\Phi$ is the multiset where the set $A_1$ occurs five times, $A_2$ nine times and $A_3$ thirteen times. The elements of $\delta_X(\Phi)$ are all those multisets containing one element per copy of $A_1$, $A_2$ and $A_3$ in $supp\,\Phi$. The $\varphi$ provided indeed contains five elements of $A_1$ (two copies of $x$ and three of $y$), nine elements of $A_2$ (four copies of $y$ and five of $z$), thirteen elements of $A_3$ (six copies of $a$ and seven of $b$).

As Example 18 shows, each element $\varphi$ of $\delta_X(\Phi)$ is determined by a function $\psi$ choosing for each set $A \in supp\,\Phi$ a finite number of elements $x_1^A, \dots, x_m^A$ in $A$ and $s_1^A, \dots, s_m^A$ in $S$ in such a way that $\sum_{j=1}^m s_j^A = \Phi(A)$. The function $\varphi$ maps each $x_j^A$ to $s_j^A$ if the sets in $supp\,\Phi$ are *disjoint*; if however there are $x_j^A$ and $x_k^B$ such that $x_j^A = x_k^B$ (like $y$ in Example 18), then $x_j^A$ is mapped to $s_j^A + s_k^B$.

Among those $\psi$'s, there are some special, *minimal* ones as it were, that choose for each $A$ in $supp\,\Phi$ exactly *one* element of $A$, and assign to it $\Phi(A)$. The induced $\varphi$ in $\delta_X(\Phi)$ can be described as $\sum_{A \in u^{-1}\{x\}} \Phi(A)$ (equivalently $\mathcal{S}(u)(\Phi)$[1]) where $u\colon supp\,\Phi \to X$ is a function selecting an element of $A$ for each $A \in supp\,\Phi$ (that is $u(A) \in A$). We denote the set of such $\varphi$'s by $\mathfrak{c}(\Phi)$.

$$\mathfrak{c}(\Phi) = \{\mathcal{S}(u)(\Phi) \mid u\colon supp\,\Phi \to X \text{ such that } \forall A \in supp\,\Phi.\, u(A) \in A\} \qquad (7)$$

*Example 20.* Take $X$, $A_1$ and $A_2$ as in Example 18, but a different, smaller, $\Phi \in \mathcal{S}(\mathcal{P}X)$ defined as $\Phi = (A_1 \mapsto 1, \quad A_2 \mapsto 2)$. There are only four functions $u\colon supp\,\Phi \to X$ such that $u(A) \in A$ and thus only four functions $\varphi$ in $\mathfrak{c}(\Phi)$:

$$\begin{array}{l|l} u_1 = (A_1 \mapsto x, \quad A_2 \mapsto y) & \varphi_1 = (x \mapsto 1,\ y \mapsto 2) \\ u_2 = (A_1 \mapsto x, \quad A_2 \mapsto z) & \varphi_2 = (x \mapsto 1,\ z \mapsto 2) \\ u_3 = (A_1 \mapsto y, \quad A_2 \mapsto y) & \varphi_3 = (y \mapsto 3) \\ u_4 = (A_1 \mapsto y, \quad A_2 \mapsto z) & \varphi_4 = (y \mapsto 1,\ z \mapsto 2) \end{array}$$

Observe that the function $\varphi = (x \mapsto 1, y \mapsto 1, z \mapsto 1)$ belongs to $\delta_X(\Phi)$ but not to $\mathfrak{c}(\Phi)$. Nevertheless $\varphi$ can be retrieved as the convex combination $\frac{1}{2} \cdot \varphi_1 + \frac{1}{2} \cdot \varphi_2$.

[1] More precisely, we should write $\mathcal{S}(u)(\Phi')$ where $\Phi'$ is the restriction of $\Phi$ to $supp\,\Phi$.

Our key result states that every $\varphi \in \delta_X(\Phi)$ can be written as a convex combination (performed in the $\mathcal{S}$-algebra $(\mathcal{S}X, \mu^{\mathcal{S}}_X)$) of functions in $\mathfrak{c}(\Phi)$, at least when $S$ is a positive semifield, which by Remark 9 and Proposition 10 satisfies all the conditions that make (6) a weak distributive law. The proof is laborious and omitted here: we only remark that divisions in $S$ play a crucial role in it.

**Theorem 21.** *Let $S$ be a positive semifield. Then for all sets $X$ and $\Phi \in \mathcal{S}\mathcal{P}X$*

$$\delta_X(\Phi) = \left\{ \mu^{\mathcal{S}}_X(\Psi) \mid \Psi \in \mathcal{S}^2 X. \sum_{\varphi \in \mathcal{S}X} \Psi(\varphi) = 1,\ supp\,\Psi \subseteq \mathfrak{c}(\Phi) \right\} = \overline{\mathfrak{c}(\Phi)}^{\mu^{\mathcal{S}}_X}. \quad (8)$$

*Remark 22.* If we drop the hypothesis of semifield and only have the minimal assumptions of Theorem 17, then (8) does not hold any more: $S = \mathbb{N}$ is a counterexample. Indeed, in this case every subset of $\mathcal{S}X$ is convex with respect to $\mu^{\mathcal{S}}_X$ (see Example 15), therefore we would have $\delta_X(\Phi) = \mathfrak{c}(\Phi)$, which is false: the function $\varphi$ of Example 18 is an example of an element in $\delta_X(\Phi) \setminus \mathfrak{c}(\Phi)$.

*Remark 23.* When $S = \mathbb{B}\text{ool}$ (which is a positive semifield), the monad $\mathcal{S}$ coincides with the monad $\mathcal{P}_f$. The function $\mathfrak{c}(\cdot)$ in (7) can then be described as

$$\mathfrak{c}(\mathcal{A}) = \{\mathcal{P}_f(u)(\mathcal{A}) \mid u \colon \mathcal{A} \to X \text{ such that } \forall A \in \mathcal{A}.\, u(A) \in A\}$$

for all $\mathcal{A} \in \mathcal{P}_f\mathcal{P}X$. It is worth remarking that this is the transformation $\chi$ appearing in Example 9 of [27] (which is in turn equivalent to the one in Example 2.4.7 of [31]). This transformation was erroneously supposed to be a distributive law, as it fails to be natural (see [28]). However, by taking its convex closure, as displayed in (8), one can turn it into a *weak* distributive law.

## 5 The Weak Lifting of $\mathcal{P}$ to $\mathbb{EM}(\mathcal{S})$

By exploiting the characterisation of the weak distributive law $\delta$ (Theorem 21), we can now describe the weak lifting of $\mathcal{P}$ to $\mathbb{EM}(\mathcal{S})$ generated by $\delta$.

Recall from Definition 13 that $\mathcal{P}^a_c X$ is the set of convex subsets of $X$ with respect to the $\mathcal{S}$-algebra $a \colon \mathcal{S}X \to X$. The functions $\iota_{(X,a)} \colon \mathcal{P}^a_c X \to \mathcal{P}X$ and $\pi_{(X,a)} \colon \mathcal{P}X \to \mathcal{P}^a_c X$ are defined for all $A \in \mathcal{P}^a_c X$ and $B \in \mathcal{P}X$ as

$$\iota_{(X,a)}(A) = A \qquad \text{and} \qquad \pi_{(X,a)}(B) = \overline{B}^a, \quad (9)$$

that is $\iota_{(X,a)}$ is just the obvious set inclusion and $\pi_{(X,a)}$ performs the convex closure in $a$. The function $\alpha_a \colon \mathcal{S}\mathcal{P}^a_c X \to \mathcal{P}^a_c X$ is defined for all $\Phi \in \mathcal{S}\mathcal{P}^a_c X$ as

$$\alpha_a(\Phi) = \{a(\varphi) \mid \varphi \in \mathfrak{c}(\Phi)\}. \quad (10)$$

To be completely formal, above we should have written $\mathfrak{c}(\mathcal{S}(\iota)(\Phi))$ in place of $\mathfrak{c}(\Phi)$, but it is immediate to see that the two sets coincide. Proving that $\alpha_a \colon \mathcal{S}\mathcal{P}^a_c X \to \mathcal{P}^a_c X$ is well defined (namely, $\alpha_a(\Phi)$ is a convex set) and forms an $\mathcal{S}$-algebra requires some ingenuity and will be shown later in Section 5.1. The assignment $(X,a) \mapsto (\mathcal{P}_c^a X, \alpha_a)$ gives rise to a functor $\tilde{\mathcal{P}} \colon \mathbb{EM}(\mathcal{S}) \to \mathbb{EM}(\mathcal{S})$ defined on morphisms $f \colon (X,a) \to (X',a')$ as

$$\tilde{\mathcal{P}}(f)(A) = \mathcal{P}f(A) \tag{11}$$

for all $A \in \mathcal{P}_c^a X$. For all $(X,a)$ in $\mathbb{EM}(\mathcal{S})$, $\eta^{\tilde{\mathcal{P}}}_{(X,a)} \colon (X,a) \to \tilde{\mathcal{P}}(X,a)$ and $\mu^{\tilde{\mathcal{P}}}_{(X,a)} \colon \tilde{\mathcal{P}}\tilde{\mathcal{P}}(X,a) \to \tilde{\mathcal{P}}(X,a)$ are defined for $x \in X$ and $\mathcal{A} \in \mathcal{P}_c^{\alpha_a}(\mathcal{P}_c^a X)$ as

$$\eta^{\tilde{\mathcal{P}}}_{(X,a)}(x) = \{x\} \qquad \text{and} \qquad \mu^{\tilde{\mathcal{P}}}_{(X,a)}(\mathcal{A}) = \bigcup_{A \in \mathcal{A}} A. \tag{12}$$

**Theorem 24.** *Let $\mathcal{S}$ be a positive semifield. Then the canonical weak lifting of the powerset monad $\mathcal{P}$ to $\mathbb{EM}(\mathcal{S})$, determined by* (8)*, consists of the monad $(\tilde{\mathcal{P}}, \eta^{\tilde{\mathcal{P}}}, \mu^{\tilde{\mathcal{P}}})$ on $\mathbb{EM}(\mathcal{S})$ defined as in* (10)*,* (11)*,* (12) *and the natural transformations $\iota \colon U^{\mathcal{S}}\tilde{\mathcal{P}} \to \mathcal{P}U^{\mathcal{S}}$ and $\pi \colon \mathcal{P}U^{\mathcal{S}} \to U^{\mathcal{S}}\tilde{\mathcal{P}}$ defined as in* (9)*.*

It is worth spelling out the left-semimodule structure on $\mathcal{P}_c^a X$ corresponding to the $\mathcal{S}$-algebra $\alpha_a \colon \mathcal{S}\mathcal{P}_c^a X \to \mathcal{P}_c^a X$. Let us start with $\lambda \cdot^{\alpha_a} A$ for some $A \in \mathcal{P}_c^a X$. By (5), $\lambda \cdot^{\alpha_a} A = \alpha_a(\Phi)$ where $\Phi = (A \mapsto \lambda)$. By (10), $\alpha_a(\Phi) = \{a(\varphi) \mid \varphi \in \mathfrak{c}(\Phi)\}$. Following the definition of $\mathfrak{c}(\Phi)$ given in (7), one has to consider functions $u \colon \mathit{supp}\, \Phi \to X$ such that $u(B) \in B$ for all $B \in \mathit{supp}\, \Phi$: if $\lambda \neq 0$, then $\mathit{supp}\, \Phi = \{A\}$ and thus, for each $x \in A$, there is exactly one function $u_x \colon \mathit{supp}\, \Phi \to X$ mapping $A$ into $x$. It is immediate to see that $\mathcal{S}(u_x)(\Phi)$ is exactly the function $(x \mapsto \lambda)$ and thus $a(\mathcal{S}(u_x)(\Phi))$ is, by (5), $\lambda \cdot^a x$. Now if $\lambda = 0$, then $\mathit{supp}\, \Phi = \emptyset$, so there is *exactly one* function $u \colon \mathit{supp}\, \Phi \to X$ and $\mathcal{S}(u)(\Phi)$ is the function mapping all $x \in X$ into 0 and thus, by (5), $a(\mathcal{S}(u)(\Phi)) = 0^a$. Summarising,

$$\lambda \cdot^{\alpha_a} A = \begin{cases} \{\lambda \cdot^a x \mid x \in A\} & \text{if } \lambda \neq 0 \\ \{0^a\} & \text{if } \lambda = 0 \end{cases} \tag{13}$$

Following similar lines of thoughts, one can check that

$$A +^{\alpha_a} B = \{x +^a y \mid x \in A,\ y \in B\} \qquad \text{and} \qquad 0^{\alpha_a} = \{0^a\}. \tag{14}$$

*Remark 25.* By comparing (14) and (13) with (4) and (5) in [25], it is immediate to see that our monad $\tilde{\mathcal{P}}$ coincides with a slight variation of Jacobs's convex powerset monad $\mathcal{C}$, the only difference being that we do allow for $\emptyset$ to be in $\mathcal{P}_c^a X$. Jacobs insisted on the necessity of $\mathcal{C}(X)$ to be the set of *non-empty* convex subsets of $X$, because otherwise he was not able to define a semimodule structure on $\mathcal{C}(X)$ such that $0 \cdot \emptyset = \{0^a\}$. However, we do manage to do so, since by (13), $0 \cdot A = 0^a$ for all $A$ and in particular for $A = \emptyset$. At first sight, this may look like an ad-hoc solution, but this is not the case: it is intrinsic in the definition of the unique weak lifting of $\mathcal{P}$ to $\mathbb{EM}(\mathcal{S})$, as stated by Theorem 24 and shown next.

### 5.1 Proof of Theorem 24

By Theorem 5, the weak distributive law (6) corresponds to a weak lifting $\tilde{\mathcal{P}}$ of $\mathcal{P}$ to $\mathbb{EM}(\mathcal{S})$, which we are going to show coincides with the data of (9)-(12). The image along $\tilde{\mathcal{P}}$ of a $\mathcal{S}$-algebra $(X, a)$ will be a set $Y$ together with a structure map $\alpha_a$ that makes it a $\mathcal{S}$-algebra in turn. Garner [15, Proposition 13] gives us the recipe to build $Y$ and $\alpha_a$ appropriately. $Y$ is obtained by splitting the following idempotent in $\mathbb{S}\mathrm{et}$:

$$e_{(X,a)} = \; \mathcal{P}X \xrightarrow{\eta^{\mathcal{S}}_{\mathcal{P}X}} \mathcal{S}(\mathcal{P}X) \xrightarrow{\delta_X} \mathcal{P}(\mathcal{S}X) \xrightarrow{\mathcal{P}a} \mathcal{P}X \tag{15}$$

as a composite $e_{(X,a)} = \iota_{(X,a)} \circ \pi_{(X,a)}$, where $\pi_{(X,a)}$ is the corestriction of $e_{(X,a)}$ to its image and $\iota_{(X,a)}$ is the set-inclusion of the image of $e_{(X,a)}$ into $\mathcal{P}X$. In other words, $Y$ is the set of fixed points of $e_{(X,a)}$. $\alpha_a$ is obtained as the composite

$$\alpha_a = \; \mathcal{S}Y \xrightarrow{\mathcal{S}\iota_{(X,a)}} \mathcal{S}\mathcal{P}X \xrightarrow{\delta_X} \mathcal{P}\mathcal{S}X \xrightarrow{\mathcal{P}a} \mathcal{P}X \xrightarrow{\pi_{(X,a)}} Y.$$

Let us, then, fix an $\mathcal{S}$-algebra $(X, a)$. Given $A \in \mathcal{P}X$, we have $\eta^{\mathcal{S}}_{\mathcal{P}X}(A) = \Delta_A \colon \mathcal{P}X \to S$, the Dirac-function centred in $A$. The set $\delta_X(\eta^{\mathcal{S}}_{\mathcal{P}X}(A))$ has a simple description, shown in the next Lemma.

**Lemma 26.** *For all $A \in \mathcal{P}X$*

$$\delta_X(\eta^{\mathcal{S}}_{\mathcal{P}X}(A)) = \left\{ \varphi \in \mathcal{S}X \mid \mathit{supp}\, \varphi \subseteq A, \sum_{x \in X} \varphi(x) = 1 \right\}.$$

The image along $A$ of the idempotent $e$ is therefore

$$e(A) = \mathcal{P}a(\delta_X(\eta^{\mathcal{S}}_{\mathcal{P}X}(A))) = \left\{ a(\varphi) \mid \varphi \in \mathcal{S}X, \mathit{supp}\, \varphi \subseteq A, \sum_{x \in X} \varphi(x) = 1 \right\} = \overline{A}^{a}.$$

Hence the idempotent $e$ computes the convex closure of elements of $\mathcal{P}X$ and its fixed points are precisely the convex subsets of $X$ with respect to the structure map $a$. Therefore, the carrier set of $\tilde{\mathcal{P}}(X, a)$ is precisely $\mathcal{P}^a_c X$, the natural transformations $\pi$ and $\iota$ are, respectively, the convex closure operator and the set-inclusion of $\mathcal{P}^a_c X$ into $\mathcal{P}X$ as in (9).

$\mathcal{P}^a_c X$ is then equipped with a structure map $\alpha_a \colon \mathcal{S}\mathcal{P}^a_c X \to \mathcal{P}^a_c X$ given by

$$\alpha_a = \; \mathcal{S}\mathcal{P}^a_c X \xrightarrow{\mathcal{S}\iota_{(X,a)}} \mathcal{S}\mathcal{P}X \xrightarrow{\delta_X} \mathcal{P}\mathcal{S}X \xrightarrow{\mathcal{P}a} \mathcal{P}X \xrightarrow{\pi_{(X,a)}} \mathcal{P}^a_c X.$$

Let us try to calculate $\alpha_a$: given $\Phi \colon \mathcal{P}^a_c X \to S$ with finite support, we have that $\mathcal{S}(\iota_{(X,a)})(\Phi)$ is just the extension of $\Phi$ to $\mathcal{P}X$ which assigns 0 to each non-convex subset of $X$. If we write $\iota$ instead of $\iota_{(X,a)}$ for short, we have

$$\alpha_a(\Phi) = \overline{\mathcal{P}a(\delta_X(\mathcal{S}(\iota)(\Phi)))}^{a}. \tag{16}$$

Next, we can use the following technical result.

**Proposition 27.** *Let $(X, a)$ be a $\mathcal{S}$-algebra. If $\mathcal{A}$ is a convex subset of $(\mathcal{S}X, \mu^{\mathcal{S}}_X)$, then $\mathcal{P}a(\mathcal{A})$ is convex in $(X, a)$.*

Since $\delta_X(\Phi')$ is the convex closure of $\mathfrak{c}(\Phi')$ in $(\mathcal{S}X, \mu^{\mathcal{S}}_X)$ for every $\Phi' \in \mathcal{SP}X$, by Proposition 27 we can avoid to perform the $a$-convex closure in (16). Therefore

$$\alpha_a(\Phi) = \mathcal{P}a(\delta_X(\mathcal{S}(\iota)(\Phi))) = \mathcal{P}a\big(\overline{\mathfrak{c}(\mathcal{S}(\iota)(\Phi))}^{\mu^{\mathcal{S}}_X}\big).$$

In the next Proposition we show that also the $\mu^{\mathcal{S}}_X$-convex closure is superfluous, due to the fact that $\Phi \in \mathcal{SP}^a_c X$ (and not simply $\mathcal{SP}X$), thus obtaining (10).

**Proposition 28.** *Let $S$ be a positive semifield, $(X, a)$ a $\mathcal{S}$-algebra, $\Phi \in \mathcal{SP}^a_c X$. Then $\mathcal{P}a(\delta_X(\mathcal{S}(\iota)(\Phi))) = \mathcal{P}a(\mathfrak{c}(\mathcal{S}(\iota)(\Phi)))$.*

*Proof.* In this proof we shall simply write $\Phi$ instead of the more verbose $\mathcal{S}(\iota)(\Phi)$. We want to prove that

$$\mathcal{P}a\big(\delta_X(\Phi)\big) = \Big\{a(\psi) \mid \psi \in \mathcal{S}X.\, \exists u \colon \mathit{supp}\,\Phi \to X.\, u(A) \in A,\ \forall x \in X.\, \psi(x) = \sum_{\substack{A \in \mathit{supp}\,\Phi \\ u(A)=x}} \Phi(A)\Big\} \quad (17)$$

where we have, by Theorem 21, that

$$\mathcal{P}a\big(\delta_X(\Phi)\big) = \{a(\mu^{\mathcal{S}}_X(\Psi)) \mid \Psi \in \mathcal{S}^2 X,\ \sum_{\varphi \in \mathcal{S}X} \Psi(\varphi) = 1,\ \mathit{supp}\,\Psi \subseteq \mathfrak{c}(\Phi)\}.$$

First of all, $\emptyset$ is *not* a $\mathcal{S}$-algebra, because there is no map $\mathcal{S}(\emptyset) \to \emptyset$ given that $\mathcal{S}(\emptyset) = \{\emptyset \colon \emptyset \to S\}$, hence $X \neq \emptyset$. Next, if $\Phi = \varepsilon \colon \mathcal{P}X \to S$, namely the function constant to 0, then $\mathfrak{c}(\Phi) = \{\varepsilon \colon X \to S\}$ therefore one can easily see that the left-hand side of (17) is equal to $\{a(\varepsilon \colon X \to S)\}$. For the same reason, the right-hand side is also equal to $\{a(\varepsilon \colon X \to S)\}$. Moreover, if $\Phi(\emptyset) \neq 0$, then there is no $u \colon \mathit{supp}\,\Phi \to X$ such that $u(\emptyset) \in \emptyset$, so $\mathfrak{c}(\Phi) = \emptyset$ and so is the left-hand side of (17); for the same reason, also the right-hand side is empty.

Suppose then, for the rest of the proof, that $\Phi \neq 0$ and that $\Phi(\emptyset) = 0$.

For the right-to-left inclusion in (17): given $\psi \in \mathfrak{c}(\Phi)$, consider $\Psi = \eta^{\mathcal{S}}_{\mathcal{S}X}(\psi) = \Delta_\psi \in \mathcal{S}^2 X$. Then $\Psi$ clearly satisfies all the required properties and $\mu^{\mathcal{S}}_X(\Psi) = \psi$.

The left-to-right inclusion is more laborious. Let $\Psi \in \mathcal{S}^2 X$ be such that $\sum_{\chi \in \mathcal{S}X} \Psi(\chi) = 1$ and such that $\mathit{supp}\,\Psi \subseteq \mathfrak{c}(\Phi)$, that is, for all $\varphi \in \mathit{supp}\,\Psi$ there is $u^\varphi \colon \mathit{supp}\,\Phi \to X$ such that $u^\varphi(A) \in A$ for all $A \in \mathit{supp}\,\Phi$ and $\varphi = \mathcal{S}(u^\varphi)(\Phi)$. We have to show that $a(\mu(\Psi)) = a(\psi)$ for some $\psi \in \mathcal{S}X$ of the form $\sum_{A \in \mathit{supp}\,\Phi} \Phi(A) \cdot u(A)$ for some choice function $u \colon \mathit{supp}\,\Phi \to X$. Notice that the given $\Psi$ is a convex linear combination of functions $\varphi$'s in $\mathcal{S}X$ like the one we have to produce: the trick will be to exploit the fact that each $A \in \mathit{supp}\,\Phi$ is convex. Here we shall only give a sketch of the proof. Suppose $\mathit{supp}\,\Phi = \{A_1, \dots, A_n\}$ and $\mathit{supp}\,\Psi = \{\varphi^1, \dots, \varphi^m\}$. Call $u^j$ the choice function that generates $\varphi^j$. Then $\Psi$ is of this form:

$$\Psi = \left( \underbrace{\begin{pmatrix} u^1(A_1) \mapsto \Phi(A_1) \\ \vdots \\ u^1(A_n) \mapsto \Phi(A_n) \end{pmatrix}}_{\varphi^1} \mapsto \Psi(\varphi^1),\ \dots,\ \underbrace{\begin{pmatrix} u^m(A_1) \mapsto \Phi(A_1) \\ \vdots \\ u^m(A_n) \mapsto \Phi(A_n) \end{pmatrix}}_{\varphi^m} \mapsto \Psi(\varphi^m) \right)$$

Define the following element of $\mathcal{S}^2 X$:

$$\Psi' = \Bigg( \underbrace{\begin{pmatrix} u^1(A_1) \mapsto \Psi(\varphi^1) \\ \vdots \\ u^m(A_1) \mapsto \Psi(\varphi^m) \end{pmatrix}}_{\chi^1} \mapsto \Phi(A_1),\ \ldots,\ \underbrace{\begin{pmatrix} u^1(A_n) \mapsto \Psi(\varphi^1) \\ \vdots \\ u^m(A_n) \mapsto \Psi(\varphi^m) \end{pmatrix}}_{\chi^n} \mapsto \Phi(A_n) \Bigg)$$

Observe that $u^1(A_i), \ldots, u^m(A_i) \in A_i$ by definition, and $A_i$ is convex by assumption: since $\sum_{j=1}^{m} \Psi(\varphi^j) = 1$, we have that $a(\chi^i) \in A_i$. Set then $u(A_i) = a(\chi^i)$ and define $\psi = \mathcal{S}(a)(\Psi')$: we have $\psi \in \mathfrak{c}(\Phi)$ with $u$ as the generating choice function. It is not difficult to see that $\mu^{\mathcal{S}}_X(\Psi) = \mu^{\mathcal{S}}_X(\Psi')$, therefore we have

$$a(\psi) = a\big(\mathcal{S}(a)(\Psi')\big) = a\big(\mu^{\mathcal{S}}_X(\Psi')\big) = a\big(\mu^{\mathcal{S}}_X(\Psi)\big)$$

as desired. $\square$

The rest of the proof of Theorem 24, concerning the action of $\tilde{\mathcal{P}}$ on morphisms and the unit and multiplication of the monad $\tilde{\mathcal{P}}$, consists in following the recipe provided by Garner [15].

## 6 The Composite Monad: an Algebraic Presentation

We can now compose the two monads $\mathcal{P}$ and $\mathcal{S}$ by considering the monad arising from the composition of the following two adjunctions:

$$\mathbb{Set} \underset{U^{\mathcal{S}}}{\overset{F^{\mathcal{S}}}{\rightleftarrows}} \mathbb{EM}(\mathcal{S}) \underset{U^{\tilde{\mathcal{P}}}}{\overset{F^{\tilde{\mathcal{P}}}}{\rightleftarrows}} \mathbb{EM}(\tilde{\mathcal{P}}) \qquad (F^{\mathcal{S}} \dashv U^{\mathcal{S}},\ F^{\tilde{\mathcal{P}}} \dashv U^{\tilde{\mathcal{P}}})$$

Direct calculations show that the resulting endofunctor on $\mathbb{Set}$, which we call $\mathcal{P}_c\mathcal{S}$, maps a set $X$ and a function $f\colon X \to Y$ into, respectively,

$$\mathcal{P}_c\mathcal{S}X = \mathcal{P}_c^{\mu^{\mathcal{S}}_X}(\mathcal{S}X) \qquad \text{and} \qquad \mathcal{P}_c\mathcal{S}(f)(\mathcal{A}) = \{\mathcal{S}(f)(\Phi) \mid \Phi \in \mathcal{A}\} \tag{18}$$

for all $\mathcal{A} \in \mathcal{P}_c\mathcal{S}X$. For all sets $X$, $\eta^{\mathcal{P}_c\mathcal{S}}_X\colon X \to \mathcal{P}_c\mathcal{S}X$ and $\mu^{\mathcal{P}_c\mathcal{S}}_X\colon \mathcal{P}_c\mathcal{S}\mathcal{P}_c\mathcal{S}X \to \mathcal{P}_c\mathcal{S}X$ are defined as

$$\eta^{\mathcal{P}_c\mathcal{S}}_X(x) = \{\Delta_x\} \qquad \text{and} \qquad \mu^{\mathcal{P}_c\mathcal{S}}_X(\mathscr{A}) = \bigcup_{\Omega \in \mathscr{A}} \alpha_{\mu^{\mathcal{S}}_X}(\Omega) \tag{19}$$

for all $x \in X$ and $\mathscr{A} \in \mathcal{P}_c\mathcal{S}\mathcal{P}_c\mathcal{S}X$.

**Theorem 29.** *Let $\mathcal{S}$ be a positive semifield. Then the canonical weak distributive law $\delta\colon \mathcal{S}\mathcal{P} \to \mathcal{P}\mathcal{S}$ given in Theorem 21 induces a monad $\mathcal{P}_c\mathcal{S}$ on* $\mathbb{Set}$ *with endofunctor, unit and multiplication defined as in* (18) *and* (19)*.*

Recall from Remark 25 that the monad $\mathcal{C}\colon \mathbb{EM}(\mathcal{S}) \to \mathbb{EM}(\mathcal{S})$ from [25] coincides with our lifting $\tilde{\mathcal{P}}$ modulo the absence of the empty set. The same happens for the composite monad, which is named $\mathcal{CM}$ in [25]. The absence of $\emptyset$ in $\mathcal{CM}$ turns out to be rather problematic for Jacobs. Indeed, in order to use the standard framework of coalgebraic trace semantics [20], one would need the Kleisli category $\mathbb{Kl}(\mathcal{CM})$ to be enriched over $\mathbb{CPPO}$, the category of $\omega$-complete partial orders with *bottom* and continuous functions. $\mathbb{Kl}(\mathcal{CM})$ is not $\mathbb{CPPO}$-enriched since there is no bottom element in $\mathcal{CM}(X)$. Instead, in $\mathcal{P}_c\mathcal{S}X$ the bottom is exactly the empty set; moreover, $\mathbb{Kl}(\mathcal{P}_c\mathcal{S})$ enjoys the properties required by [20].

**Theorem 30.** *The category $\mathbb{Kl}(\mathcal{P}_c\mathcal{S})$ is enriched over $\mathbb{CPPO}$ and satisfies the left-strictness condition: for all $f\colon X \to \mathcal{P}_c\mathcal{S}Y$ and $Z$ set, $\bot_{Y,Z} \circ f = \bot_{X,Z}$.*

It is immediate that every homset in $\mathbb{Kl}(\mathcal{P}_c\mathcal{S})$ carries a complete partial order. Showing that composition of arrows in $\mathbb{Kl}(\mathcal{P}_c\mathcal{S})$ preserves joins (of $\omega$-chains) requires more work: the proof, omitted here, crucially relies on the algebraic theory presenting the monad $\mathcal{P}_c\mathcal{S}$, illustrated next.

**An Algebraic Presentation.** Recall that an *algebraic theory* is a pair $\mathcal{T} = (\Sigma, E)$ where $\Sigma$ is a *signature*, whose elements are called *operations*, to each of which is assigned a cardinal number called its *arity*, while $E$ is a class of formal *equations* between $\Sigma$-terms. An *algebra* for the theory $\mathcal{T}$ is a set $A$ together with, for each operation $o$ of arity $\kappa$ in $\Sigma$, a function $o_A\colon A^\kappa \to A$ satisfying the equations of $E$. A *homomorphism* of algebras is a function $f\colon A \to B$ respecting the operations of $\Sigma$ in their realisations in $A$ and $B$. Algebras and homomorphisms of an algebraic theory $\mathcal{T}$ form a category $\mathbb{Alg}(\mathcal{T})$.

**Definition 31.** *Let $M$ be a monad on* $\mathbb{Set}$*, and $\mathcal{T}$ an algebraic theory. We say that $\mathcal{T}$* presents *$M$ if and only if $\mathbb{EM}(M)$ and $\mathbb{Alg}(\mathcal{T})$ are isomorphic.*

Left $S$-semimodules are algebras for the theory $\mathcal{LSM} = (\Sigma_{\mathcal{LSM}}, E_{\mathcal{LSM}})$ where $\Sigma_{S\mathcal{LSM}} = \{+, 0\} \cup \{\lambda\cdot \mid \lambda \in S\}$ and $E_{\mathcal{LSM}}$ is the set of axioms in Table 1. As already mentioned in Section 3, left $S$-semimodules are exactly $\mathcal{S}$-algebras and morphisms of $S$-semimodules coincide with those of $\mathcal{S}$-algebras. Thus, the theory $\mathcal{LSM}$ presents the monad $\mathcal{S}$.

Similarly, semilattices are algebras for the theory $\mathcal{SL} = (\Sigma_{\mathcal{SL}}, E_{\mathcal{SL}})$ where $\Sigma_{\mathcal{SL}} = \{\sqcup, \bot\}$ and $E_{\mathcal{SL}}$ is the set of axioms in Table 1. It is well known that semilattices are algebras for the *finite* powerset monad. Actually, this monad is presented by $\mathcal{SL}$. In order to present the full powerset monad $\mathcal{P}$ we need to take joins of arbitrary arity. A *complete semilattice* is a set $X$ equipped with joins $\bigsqcup_{x\in A} x$ for all–not necessarily finite–$A \subseteq X$. Formally the (infinitary) theory of *complete semilattices* is given as $\mathcal{CSL} = (\Sigma_{\mathcal{CSL}}, E_{\mathcal{CSL}})$ where $\Sigma_{\mathcal{CSL}} = \{\bigsqcup_I \mid I \text{ set}\}$ and $E_{\mathcal{CSL}}$ is the set of axioms displayed in Table 3 (for a detailed treatment of infinitary algebraic theories see, for example, [30]).

We can now illustrate the theory $(\Sigma, E)$ presenting the composed monad $\mathcal{P}_c\mathcal{S}$: the operations in $\Sigma$ are exactly those of complete semilattices and $S$-

**Table 3.** The sets of axioms $E_{\mathcal{CSL}}$ for complete semilattices: the second axiom generalises the usual idempotency and commutativity properties of finitary $\sqcup$, while the third one generalises associativity and neutrality of $\bigsqcup_{\emptyset} = \bot$.

$$\bigsqcup_{i\in\{0\}} x_i = x_0$$
$$\bigsqcup_{j\in J} x_j = \bigsqcup_{i\in I} x_{f(i)} \text{ for all } f\colon I \to J \text{ surjective}$$
$$\bigsqcup_{i\in I} x_i = \bigsqcup_{j\in J} \bigsqcup_{i\in f^{-1}\{j\}} x_i \text{ for all } f\colon I \to J$$

semimodules, while the axioms are those of complete semilattices and $S$-semimodules together with the set $E_{\mathcal{D}}$ of *distributivity* axioms illustrated below.

$$\lambda \cdot \bigsqcup_{i\in I} x_i = \bigsqcup_{i\in I} \lambda \cdot x_i \quad \text{for } \lambda \neq 0, \qquad \bigsqcup_{i\in I} x_i + \bigsqcup_{j\in J} y_j = \bigsqcup_{(i,j)\in I\times J} x_i + y_j \tag{20}$$

In short, $\Sigma = \Sigma_{\mathcal{CSL}} \cup \Sigma_{\mathcal{LSM}}$ and $E = E_{\mathcal{CSL}} \cup E_{\mathcal{LSM}} \cup E_{\mathcal{D}}$.

**Theorem 32.** *The monad $\mathcal{P}_c\mathcal{S}$ is presented by the algebraic theory $(\Sigma, E)$.*

The presentation crucially relies on the fact that $\mathcal{P}_c\mathcal{S}$ is obtained by composing $\mathcal{P}$ and $\mathcal{S}$ via $\delta$. Indeed, we know from general results in [11,15] that $\mathcal{P}_c\mathcal{S}$-algebras are in one to one correspondence with $\delta$-algebras [3], namely triples $(X, a, b)$ such that $a\colon \mathcal{S}X \to X$ is a $\mathcal{S}$-algebra, $b\colon \mathcal{P}X \to X$ is a $\mathcal{P}$-algebra and the following diagram commutes.

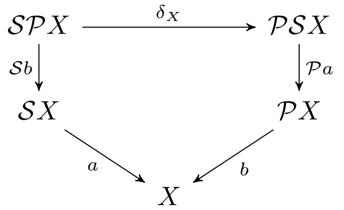

(21)

The $\mathcal{S}$-algebra $a$ corresponds to a $S$-semimodule $(X, +, 0, \lambda\cdot)$, the $\mathcal{P}$-algebra $b$ to a complete lattice $(X, \bigsqcup_I)$ and the commutativity of diagram (21) expresses exactly the distributivity axioms in (20).

*Example 33.* Let $S$ be $\mathbb{R}^+$ and let $[a, b]$ with $a, b \in \mathbb{R}^+$ denote the set $\{x \in \mathbb{R}^+ \mid a \leq x \leq b\}$ and $[a, \infty)$ the set $\{x \in \mathbb{R}^+ \mid a \leq x\}$. For $1 = \{x\}$, $\mathcal{P}_c\mathcal{S}(1) = \{\emptyset\} \cup \{[a, b] \mid a, b \in \mathbb{R}^+\} \cup \{[a, +\infty) \mid a \in \mathbb{R}^+\}$. The $\mathcal{P}_c\mathcal{S}$-algebra $\mu_1^{\mathcal{P}_c\mathcal{S}}\colon \mathcal{P}_c\mathcal{S}\mathcal{P}_c\mathcal{S}1 \to \mathcal{P}_c\mathcal{S}1$ induces a $\delta$-algebra where the structure of complete lattice is given as[2]

$$\bigsqcup_{i\in I} A_i = \begin{cases} [\inf_{i\in I}, a_i, \sup_{i\in I} b_i] & \text{if, for all } i \in I,\ A_i = [a_i, b_i] \wedge \sup_{i\in I} b_i \in \mathbb{R}^+ \\ [\inf_{i\in I} a_i, \infty) & \text{otherwise} \end{cases}$$

The $\mathbb{R}^+$-semimodule is as expected, e.g., $[a_1, b_1] + [a_2, b_2] = [a_1 + a_2, b_1 + b_2]$.

[2] For the sake of brevity, we are ignoring the case where some $A_i = \emptyset$.

**Finite Joins and Finitely Generated Convex Sets.** We now consider the algebraic theory $(\Sigma', E')$ obtained by restricting $(\Sigma, E)$ to finitary joins. More precisely, we fix

$$\Sigma' = \Sigma_{\mathcal{SL}} \cup \Sigma_{\mathcal{LSM}} \qquad E' = E_{\mathcal{SL}} \cup E_{\mathcal{LSM}} \cup E_{\mathcal{D}'}$$

where $(\Sigma_{\mathcal{SL}}, E_{\mathcal{SL}})$ is the algebraic theory for semilatices, $(\Sigma_{\mathcal{LSM}}, E_{\mathcal{LSM}})$ is the one for $S$-semimodules, and $E_{\mathcal{D}'}$ is the set of distributivity axioms illustrated in Table 1. Thanks to the characterisation provided by Theorem 32, we easily obtain a function translating $\Sigma'$-terms into convex subsets.

**Proposition 34.** *Let $T_{\Sigma',E'}(X)$ be the set of $\Sigma'$-terms with variables in $X$ quotiented by $E'$. Let $[\![\cdot]\!]_X : T_{\Sigma',E'}(X) \to \mathcal{P}_c\mathcal{S}(X)$ be the function defined as*

$$[\![x]\!] = \{\Delta_x\} \text{ for } x \in X \qquad [\![\lambda \cdot t]\!] = \begin{cases} \{\lambda \cdot^{\mu^S} f \mid f \in [\![t]\!]\} & \text{if } \lambda \neq 0 \\ \{0^{\mu^S}\} & \text{otherwise} \end{cases}$$

$$[\![0]\!] = \{0^{\mu^S}\} \qquad [\![t_1 + t_2]\!] = \{f_1 +^{\mu^S} f_2 \mid f_1 \in [\![t_1]\!],\ f_2 \in [\![t_2]\!]\}$$

$$[\![\bot]\!] = \emptyset \qquad [\![t_1 \sqcup t_2]\!] = \overline{[\![t_1]\!] \cup [\![t_2]\!]}^{\mu^S}$$

*Let $[\![\cdot]\!] : T_{\Sigma',E'} \to \mathcal{P}_c\mathcal{S}$ be the family $\{[\![\cdot]\!]_X\}_{X \in |\mathbb{S}\mathrm{et}|}$. Then $[\![\cdot]\!] : T_{\Sigma',E'} \to \mathcal{P}_c\mathcal{S}$ is a map of monads and, moreover, each $[\![\cdot]\!]_X : T_{\Sigma',E'}(X) \to \mathcal{P}_c\mathcal{S}(X)$ is injective.*

We say that a set $\mathcal{A} \in \mathcal{P}_c\mathcal{S}(X)$ is *finitely generated* if there exists a finite set $\mathcal{B} \subseteq \mathcal{S}(X)$ such that $\overline{\mathcal{B}} = \mathcal{A}$. We write $\mathcal{P}_{fc}\mathcal{S}(X)$ for the set of all $\mathcal{A} \in \mathcal{P}_c\mathcal{S}(X)$ that are finitely generated. The assignment $X \mapsto \mathcal{P}_{fc}\mathcal{S}(X)$ gives rise to a monad $\mathcal{P}_{fc}\mathcal{S} : \mathbb{S}\mathrm{et} \to \mathbb{S}\mathrm{et}$ where the action on functions, the unit and the multiplication are defined as for $\mathcal{P}_c\mathcal{S}$.

**Theorem 35.** *The monads $T_{\Sigma',E'}$ and $\mathcal{P}_{fc}\mathcal{S}$ are isomorphic. Therefore $(\Sigma', E')$ is a presentation for the monad $\mathcal{P}_{fc}\mathcal{S}$.*

*Example 36.* Recall $\mathcal{P}_c\mathcal{S}(1)$ for $S = \mathbb{R}^+$ from Example 33. By restricting to the finitely generated convex sets, one obtains $\mathcal{P}_{fc}\mathcal{S}(1) = \{\emptyset\} \cup \{[a, b] \mid a, b \in \mathbb{R}^+\}$, that is the sets of the form $[a, \infty)$ are not finitely generated. Table 4 illustrates the isomorphism $[\![\cdot]\!] : T_{\Sigma',E'}(1) \to \mathcal{P}_c\mathcal{S}(1)$. It is worth observing that every closed interval $[a, b]$ is denoted by a term in $T_{\Sigma',E'}(1)$ for $1 = \{x\}$: indeed, $[\![(a \cdot x) \sqcup (b \cdot x)]\!] = [a, b]$. For $2 = \{x, y\}$, $\mathcal{P}_{fc}\mathcal{S}(2)$ is the set containing all convex polygons: for instance the term $(r_1 \cdot x + s_1 \cdot y) \sqcup (r_2 \cdot x + s_2 \cdot y) \sqcup (r_3 \cdot x + s_3 \cdot y)$ denote a triangle with vertexes $(r_i, s_i)$. For $n = \{x_0, \dots x_{n-1}\}$, it is easy to see that $\mathcal{P}_{fc}\mathcal{S}(n)$ contains all convex $n$-polytopes.

## 7 Conclusions: Related and Future Work

Our work was inspired by [17] where Goy and Petrisan compose the monads of powerset and probability distributions by means of a weak distributive law in the sense of Garner [15]. Our results also heavily rely on the work of Clementino

**Table 4.** The inductive definition of the function $[\![\cdot]\!]_1 \colon T_{\Sigma',E'}(1) \to \mathcal{P}_c\mathcal{S}(1)$ for $1 = \{x\}$.

$$[\![x]\!] = [1,1] \qquad [\![0]\!] = [0,0] \qquad [\![\bot]\!] = \emptyset$$

$$[\![\lambda \cdot t]\!] = \begin{cases} [\lambda \cdot a, \lambda \cdot b] & \text{if } \lambda \neq 0,\ [\![t]\!] = [a,b] \\ \emptyset & \text{if } \lambda \neq 0,\ [\![t]\!] = \emptyset \\ [0,0] & \text{otherwise} \end{cases}$$

$$[\![t_1 + t_2]\!] = \begin{cases} [a_1 + a_2, b_1 + b_2] & \text{if } [\![t_i]\!] = [a_i, b_i] \\ \emptyset & \text{otherwise} \end{cases}$$

$$[\![t_1 \sqcup t_2]\!] = \begin{cases} [min\ a_i,\ max\ b_i] & \text{if } [\![t_i]\!] = [a_i, b_i] \\ [a_1, b_1] & \text{if } [\![t_1]\!] = [a_1, b_1],\ [\![t_2]\!] = \emptyset \\ [a_2, b_2] & \text{if } [\![t_2]\!] = [a_2, b_2],\ [\![t_1]\!] = \emptyset \\ \emptyset & \text{otherwise} \end{cases}$$

et al. [12] that illustrates necessary and sufficient conditions on a semiring $S$ for the existence of a weak distributive law $\delta \colon \mathcal{SP} \to \mathcal{PS}$. However, to the best of our knowledge, the alternative characterisation of $\delta$ provided by Theorem 21 was never shown.

Such characterisation is essential for giving a handy description of the lifting $\tilde{\mathcal{P}} \colon \mathbb{EM}(\mathcal{S}) \to \mathbb{EM}(\mathcal{S})$ (Theorem 24) as well as to observe the strong relationships with the work of Jacobs (Remark 25) and the one of Klin and Rot (Remark 23). The weak distributive law $\delta$ also plays a key role in providing the algebraic theories presenting the composed monad $\mathcal{P}_c\mathcal{S}$ (Theorem 24) and its finitary restriction $\mathcal{P}_{fc}\mathcal{S}$ (Theorem 35). These two theories resemble those appearing in, respectively, [17] and [10] where the monad of probability distributions plays the role of the monad $\mathcal{S}$ in our work.

Theorem 30 allows to reuse the framework of coalgebraic trace semantics [20] for modelling over $\mathbb{Kl}(\mathcal{P}_c\mathcal{S})$ systems with both nondeterminism and quantitative features. The alternative framework based on coalgebras over $\mathbb{EM}(\mathcal{P}_c\mathcal{S})$ directly leads to *nondeterministic weighted automata*. A proper comparison with those in [13] is left as future work. Thanks to the abstract results in [7], language equivalence for such coalgebras could be checked by means of coinductive up-to techniques. It is worth remarking that, since $\delta$ is a weak distributive law, then thanks to the work in [16], up-to techniques are also sound for "convex-bisimilarity" (in coalgebraic terms, behavioural equivalence for the lifted functor $\tilde{\mathcal{P}} \colon \mathbb{EM}(\mathcal{S}) \to \mathbb{EM}(\mathcal{S})$).

We conclude by recalling that we have two main examples of positive semifields: $\mathbb{B}$ool and $\mathbb{R}^+$. Booleans could lead to a coalgebraic modal logic and trace semantics for *alternating automata* in the style of [27]. For $\mathbb{R}^+$, we hope that exploiting the ideas in [34] our monad could shed some lights on the behaviour of linear dynamical systems featuring some sort of nondeterminism.
